# Depolymerase improves gentamicin efficacy during *Klebsiella pneumoniae* induced murine infection

**DOI:** 10.1186/1471-2334-14-456

**Published:** 2014-08-23

**Authors:** Shruti Bansal, Kusum Harjai, Sanjay Chhibber

**Affiliations:** Department of Microbiology, Panjab University, Sector-14, Chandigarh, 160014 India

**Keywords:** *Klebsiella pneumoniae*, *Aeromonas punctata*, Innate immune response, Capsule depolymerase, Gentamicin

## Abstract

**Background:**

Presence of capsule enhances the virulence of bacteria that cause pneumonia, meningitis, cystic fibrosis, dental caries, periodontitis. Capsule is an important virulence factor for *Klebsiella pneumoniae* and infections due to this pathogen have been associated with high mortality rates. In the present study, use of an *Aeromonas punctata* derived capsule depolymerase against *K. pneumoniae*, to reinstate the efficacy of gentamicin during pneumonia and septicemia was investigated.

**Methods:**

Depolymerase was administered in mice intraperitoneally (50 μg) alone as well in combination with gentamicin (1.5 mg/kg), 24 h post infection during acute lung infection and 6 h later during septicemia. Bacterial load, neutrophil infiltration and cytokine levels were estimated. The immunogenicity of protein was also studied.

**Results:**

In comparison to groups treated with gentamicin alone, combination treatment with depolymerase and gentamicin significantly reduced (P < 0.01) bacterial titer in the lungs, liver, kidney, spleen and blood of experimental animals. Highly significant reduction in neutrophil infiltration and levels of pro-inflammatory and anti-inflammatory cytokines was also observed. This indicated an efficient capsule removal by the enzyme, that improved gentamicin efficacy *in vivo*. Although the enzyme was found to be immunogenic, but no significant reduction in treatment efficacy was observed in the preimmunized as well as naïve mice. In addition, as confirmed through flow cytometry, the hyperimmune sera raised against the enzyme did not neutralize its activity.

**Conclusion:**

The results confirm that administration of enzyme ‘depolymerase’ along with gentamicin not only checked the virulence of *K. pneumoniae in vivo* but it also increased its susceptibility to gentamicin at a lower concentration. Such a strategy would help to avoid exposure to higher concentration of gentamicin. Moreover, since this decapsulating protein does not possess a lytic activity therefore there would be no chances of development of bacterial resistance against it. Therefore, it should be studied further for its successful inclusion in our prophylactic/therapeutic regimes.

**Electronic supplementary material:**

The online version of this article (doi:10.1186/1471-2334-14-456) contains supplementary material, which is available to authorized users.

## Background

Capsular polysaccharide (CPS) is a discrete layer tightly bound to the bacterial cell wall. It is a major virulence determinant in pathogens like *Streptococcus pneumoniae*, *Neisseria meningitidis*, *Haemophilus influenzae*, *Klebsiella pneumoniae*, *Escherichia coli*, *Cryptococcus neoformans* etc. [1–4]. It mediates adherence, protects bacteria against dessication, bacteriophages and immune response [5]. Strains possessing capsules can evade immune responses by several mechanisms. Firstly, their hydrophilic nature and net negative charge repel the negatively charged surface of phagocytes thereby diminishing bacterial removal through phagocytosis [6]. Secondly, capsular polysaccharides mask the pathogen-associated molecular patterns (PAMPs) that are recognised by Toll-like receptors (TLRs) thereby preventing activation of immune response [7, 8]. Thirdly, presence of capsule may cause deficient binding, degradation or masking of complement components [9]. In addition, encapsulated bacteria might be protected from various antimicrobial peptides (APs) present in mucosal surfaces [10].

*Klebsiella pneumoniae* is a common cause of nosocomial infections including urinary tract, respiratory, wound infections, bacteremia, septicemia, pyogenic liver abscess etc. [11]. *K. pneumoniae* CPS is antiphagocytic, mediates serum resistance and induces cytotoxicity during infection of lung epithelial cells. It also helps in bacterial colonization and biofilm formation at infection sites [12]. Of the 78 chemically distinct capsular types of *K. pneumoniae,* K1 and K2 are the most virulent. They have been frequently isolated from patients with bacteremia and respiratory tract infections [13, 14]. Aminoglycosides and fluoroquinolones have been widely used for restricting the growth of members of Enterobacteriaceae family. Antibiotics might kill the free-floating bacteria *in vivo* but fail to eradicate bacterial cells embedded in a biofilm [15]. The, bacterial CPS is known to interfere with the penetration of many antibiotics, leading to exposure of bacteria to sub-MIC concentrations, that increases the risk of resistance [16]. These mounting concerns underscore the need for an effective alternative treatment strategy for infections.

CPS is an excellent target for compounds aimed at replacing or supplementing antibiotic treatment for microbial infections. CPS synthesis can be blocked by deleting or downregulating the genes for capsule biosynthesis [5]. The therapeutic interventions include ‘capsule-stripping’ by using naturally occurring or engineered bacteria or bacteriophages that secrete pathogen-specific CPS-depolymerizing enzymes like lyase, glycosidase, endosialidase, polyglutamic acid depolymerase [5].

No report is available on the *in vivo* use of an enzyme derived from unrelated genera capable of decapsulating *K. pneumoniae.* In the present study, a *Aeromonas punctata* derived depolymerase was used to alter the severity of infection during *K. pneumoniae* induced pneumonia and septicemia. Enzyme treatment of this kind can provide several advantages during bacterial infections. They attenuate the virulence of denuded pathogen and improve its susceptibility to immune defences. They can also be useful as adjuncts to antibiotics for an early recovery from infection. Since they are not directly bactericidal they do not allow generation of resistant mutants and because they are highly specific so they do not target normal flora [17].

## Methods

### Bacterial strains, antibiotic

*Klebsiella pneumoniae* B5055 (O1:K2), obtained from Dr Mathia Trautmann, Department of Medical Microbiology and Hygiene, University of Ulm, Germany, stored in our laboratory in 60% glycerol at -80°C and maintained on nutrient agar slants at 4°C, was used in the study. Gentamicin (Himedia, India) stock solution (2 mg/ml) was prepared in sterile distilled water according to the method of Andrews [18]. It was administered to mice at a final concentration of 1.5 mg/kg body wt. throughout the study.

### Enzyme

Bacterial depolymerase isolated from *A. punctata* (GenBank: KF158411), capable of acting on the K2 capsular polysaccharide of *K. pneumoniae* B5055 (composed of glucose, mannose and glucuronic acid) was used. Production of bacterial depolymerase was carried out by cultivating *A. punctata* in a statistically optimized media as standardized in our laboratory [19]. Cell free supernatant containing enzyme was obtained and purified by anion exchange (DEAE) followed by gel filtration chromatography (Sephadex G100). The enzyme was purified to homogeneity and used at a concentration of 50 μg throughout the study. [Decapsulation of *K. pneumoniae* B5055 after treatment with *A. punctata* derived depolymerase is depicted in Additional file 1].

### Animals

Pathogen-free BALB/c mice of either sex, 6–8 weeks old, weighing 20–25 g were procured from the central animal house of Panjab University, Chandigarh, India. Animals were kept in clear polypropylene cages and fed on a standard antibiotic-free diet (Hindustan Lever Products, Kolkata, India) and water ad libitum. The temperature ranged between 18 and 22°C and relative humidity was between 55 and 65%.

Ethics statement: The study was conducted after obtaining approval from the Institutional Animal ethics committee of Panjab University [Approval ID: IAEC/346-356]. All experiment protocols were performed in accordance with the guidelines of Committee for the Purpose of Control and Supervision of Experiments on Animals (CPCSEA), Government of India. All efforts were made to minimize the suffering of animals.

[Note: Bacterial doses corresponding to 10^2^-10^8^ CFU/ml were tried for inducing acute lung infection after intranasal administration or septicemia after intraperitoneal administration. The dose which gave 100% infection without causing any mortality was chosen for this work. (i.e. 10^4^ CFU/50 μl for i.n infection and 10^2^ CFU/100 μl for systemic infection by i.p route)].

### Treatment efficacy in acute lung infection model

*K. pneumoniae* B5055 was cultivated for 24 h at 37°C in nutrient broth. Next day, cells were pelleted and washed twice with normal saline (0.85% NaCl). Bacterial suspension prepared in saline was adjusted to achieve a cell density corresponding to 2 × 10^5^ CFU/ml (O.D = 0.03). Acute lung infection was induced in mice following the method of Held et al. [20] modified by Yadav et al. [21]. Intranasal instillation of 10^4^ CFU of *K. pneumoniae* B5055 in a volume of 50 μl was performed by holding the mice in an upright position without any anaesthesia. Infected mice were then randomly divided into four groups with each group comprising 20 animals. Different groups were given one of the following treatments:

Group 1 (control): Mice infected with *K. pneumoniae* B5055, given normal saline intraperitoneally.

Group 2: Mice infected with *K. pneumoniae* B5055 followed by intraperitoneal administration of gentamicin (1.5 mg/kg/daily), initiated 24 h post infection.

Group 3: Mice infected with *K. pneumoniae* B5055 followed by intraperitoneal administration of the *A. punctata* derived depolymerase (50 μg), 24 h post infection.

Group 4: Mice infected with *K. pneumoniae* B5055 followed by intraperitoneal administration of depolymerase (50 μg) as well as gentamicin (1.5 mg/kg, daily), 24 h post infection.

[Note: Optimal dose of *A. punctata* derived depolymerase to be used was chosen based on the results of preliminary experiments carried out using different doses, 25 μg, 50 μg and 75 μg. Dose giving maximum log reduction after *in vivo* administration was thus selected].

Animals were sacrificed on different days [1–3, 5, 7] post-infection by cervical dislocation and lungs were removed aseptically. Bacterial load, pro-inflammatory and anti-inflammatory cytokine levels were estimated in lung tissue homogenates while histopathological examination was carried out for the intact lung tissue.

### Effect on systemic infection

Systemic infection was induced in mice by intraperitoneal administration of 10^2^ CFU of overnight grown and washed *K. pneumoniae* B5055 in a volume of 0.1 ml. Thereafter, mice were divided into the following 4 groups with each group comprising of 10 animals:

Group 1 (control): Mice infected with *K. pneumoniae* B5055 and given normal saline intraperitoneally, 6 h post infection.

Group 2: Mice infected with *K. pneumoniae* B5055, administered gentamicin (1.5 mg/kg) intraperitoneally, 6 h post infection.

Group 3: Mice infected with *K. pneumoniae* B5055, administered bacterial depolymerase (50 μg), intraperitoneally, 6 h post infection.

Group 4: Mice infected with *K. pneumoniae* B5055, administered bacterial depolymerase (50 μg) and gentamicin (1.5 mg/kg), intraperitoneally, 6 h post infection.

In each group, blood was taken from mice by retro-orbital puncture and lungs, liver, kidney, spleen were removed aseptically, 24 h post infection. Estimation of bacterial load and pro and anti-inflammatory cytokine was carried out in tissue homogenates.

### Quantification of bacteria

Mice were sacrificed on different days post-infection by cervical dislocation. Different organs were removed aseptically and homogenized in 1 ml normal saline. Serial dilutions of the homogenized tissues were made and plated on nutrient agar plates. Plates were incubated at 37°C for 24 h and bacterial counts determined.

### Estimation of cytokine levels

Assay for Tumor necrosis factor *a* (TNF-*a*), Interleukin-1 (IL-1), Interleukin-10 (IL-10) was performed by ELISA using commercially available cytokine kits (Peprotech). Lungs, liver, kidney, spleen were homogenized in 1 ml lysis buffer containing 0.5% Triton X 100, 150 mM NaCl, 15 mM Tris, 1 mM CaCl_2_ and 1 mM MgCl_2_ (pH 7.4). Homogenates were centrifuged at 400 g for 10 min and supernatants were used for estimation of cytokine levels. Appropriate antigen-affinity purified anti-mouse antibody pairs, detection reagents (TMB, BD biosciences) and mouse recombinant cytokines obtained from Peprotech were used as standards (capture antibodies: rabbit anti-mouse IL-1ß/IL-10/TNF*a*; detection antibodies: biotinylated rabbit anti-mouse IL-1ß/IL-10/TNF*a*). Absorbance was measured at 450 nm and results were expressed as pg/ml of cytokines released. All assays were performed in triplicates and performed thrice.

### Histopathological examination

Lungs were removed aseptically, immersed in 10% formalin fixative and processed for histological examination. The lung tissue was embedded in paraffin wax and cut into 4–6 μm thick sections using a microtome. The sections were stained with haematoxylin and eosin and assessed for neutrophil infiltration and inflammatory changes.

### Generation of antisera

For raising antisera against bacterial depolymerase, 10 Balb/c mice were injected sub-cutaneously on day 0 with 50 μg protein emulsified in CFA (Sigma Aldrich). It was followed by subcutaneous injection with booster doses on days 7, 14 and 21 [50 μg protein emulsified in IFA (Sigma Aldrich)]. Thereafter, mice were bled through retro-orbital puncture on 10^th^, 17^th^ and 28^th^ day and blood serum was collected. Antibody titer was determined in serum samples using 1:25,000 dilution of goat anti-mouse HRP conjugate (Bangalore genei) as the secondary antibody in an enzyme linked immunosorbent assay. Antibody titer was defined as the reciprocal of dilution that gave an absorbance of 1.0 at 450 nm after a 30-min reaction with the chromogenic substrate (TMB).

#### Enzyme efficacy in vivo in presence of antisera

Mice were divided into the following 3 groups with each group comprising of 10 animals:

Group 1: Naïve control mice challenged intranasally with 10^4^ CFU of *K. pneumoniae* B5055 in a volume of 50 μl followed by treatment with normal saline (0.1 ml/i.p), 24 h post infection.

Group 2: Naïve control mice challenged intranasally with 10^4^ CFU of *K. pneumoniae* B5055 in a volume of 50 μl followed by treatment with bacterial depolymerase (50 μg, i.p), 24 h post infection.

Group 3: Immunized mice (with antibodies against depolymerase) challenged intranasally with 10^4^ CFU of *K. pneumoniae* B5055 in a volume of 50 μl followed by treatment with bacterial depolymerase (50 μg, i.p), 24 h post infection.

Bacterial count was determined on the peak day (day 3) in lungs homogenates of mice belonging to the three groups and Log_10_CFU/ml was calculated.

#### Enzyme activity following incubation with antisera

Bacterial depolymerase was preincubated at 37°C for 60 min with sera obtained from naïve and immunized mice (antibody titer: 1000) (sera was heated at 56°C for 30 min to inactivate complement before use). Following this, log phase *K. pneumoniae* (10^8^ CFU/ml) were treated with these pre-incubated enzyme samples at 37°C for 60 min. These cells were then washed twice with Hank’s balanced salt solution (HBSS; 10 mM PBS, pH 7.2, containing 1 mM CaCl_2_, 0.5 mM MgCl_2_ and 1 mg/ml glucose) and bacterial number was determined. The bacterial cells were then opsonized with 10% normal mouse serum (taken from uninfected mice) for 20 min at 37°C and bacterial number was confirmed. Thereafter, phagocytosis of bacteria by macrophages was performed by the method of Hampton and Winterbourn, [22].

For phagocytosis, the killing efficacy depends on the MOI i.e. ratio of bacteria and macrophages. In our study, we tried different MOIs i.e. 1, 10, 100, 1000. But the best results were obtained with MOI 100. Therefore, it was selected for this study. Briefly, peritoneal macrophages (10^6^/ml) were isolated from the peritoneal lavage collected from pathogen free BALB/c mice. Macrophages were suspended in HBSS containing 10% normal mouse serum. These were incubated with pretreated opsonized bacterial cells (10^8^ CFU/ml) at 37°C in 5% CO2. Samples were withdrawn at appropriate time intervals and an equal volume of ice-cold PBS was added followed by centrifugation at 200 g for 5 min. Supernatant was separated, pellet was washed twice, suspended in PBS with 0.5% triton X solution and incubated at room temperature for 30 min. Intracellular bacteria recovered after 3 h were stained with a LIVE-DEAD assay kit (Molecular Probes) consisting of 30 nM SYTO9 and 15 μM propidium iodide for 15 min in dark. Analysis was done by flow cytometry, using a flow cytometer (BD Biosciences FACS Canto II) and FACSdiva software. A control tube to study phagocytic killing of bacterial cells not treated with depolymerase was also put up simultaneously.

### Statistical analysis

Results were analysed statistically by applying one-way ANOVA (Microsoft Excel 2007) for comparing various parameters in treated and untreated control mice. Differences were considered statistically significant if P-value was less than 0.01.

## Results

### Effect of intra-peritoneal administration of bacterial depolymerase on progression of compartmentalized lung infection

**Figure 1:**
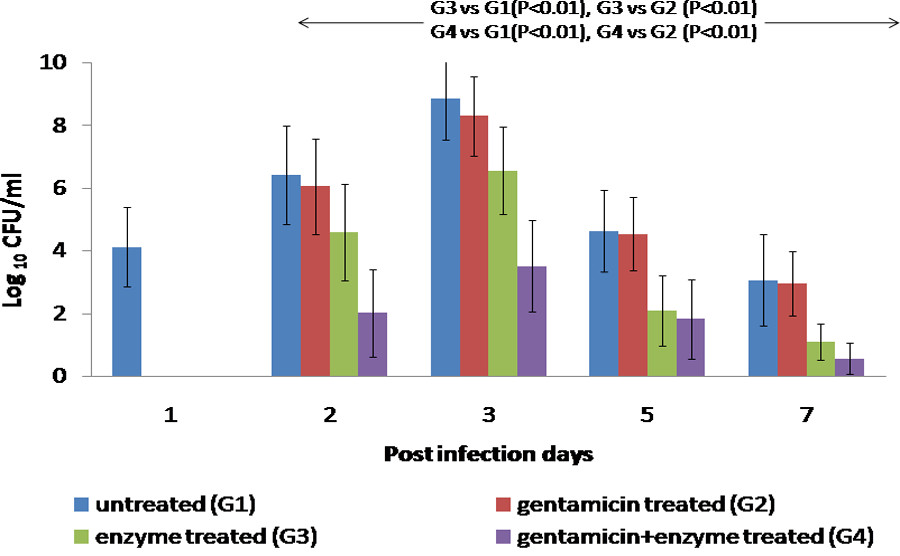
**Bacterial load (Log**_**10**_ **CFU ml** ^**-1**^ **) in the lungs of mice infected via the intranasal route with** ***K. pneumoniae*** **B5055 and treated with gentamicin (1.5 mg/kg) and/or depolymerase (50 μg).** No sample was taken and processed on day 1 in the treated groups. G1, G2, G3, G4 represent Groups 1, 2, 3, 4. Arrows indicate significant reduction in Log bacterial count between groups on days 2, 3, 5, 7. Error bars represent standard deviation (S.D) from four independent values.

Intranasal instillation of bacteria in mice resulted in bacterial load of 4.2 logs, 24 h post infection (Figure 1, day 1). It was similar to the intranasal dose administered to mice. Thereafter, multiplication of bacteria in lungs resulted in a peak in bacterial count on day 3 (8.849 ± 1.321) followed by subsequent decrease (Figure 1). Intraperitoneal injection of gentamicin daily (initiated 24 h post infection) was not effective in this model of experimental pneumonia as bacterial counts were similar to that observed in untreated infected animals throughout the course of infection. When bacterial depolymerase was injected 24 h post infection, although bacteria were present in the lung tissue throughout the course of infection (Figure 1) but, an average reduction of ~2 logs (P < 0.01) was observed. Co-administration of gentamicin along with bacterial depolymerase resulted in significant reduction (P < 0.01) in average bacterial count (~3.4 logs) in comparison to the untreated or gentamicin treated animals.

### Effect of intra-peritoneal administration of bacterial depolymerase on systemic spread of bacteria

**Figure 2:**
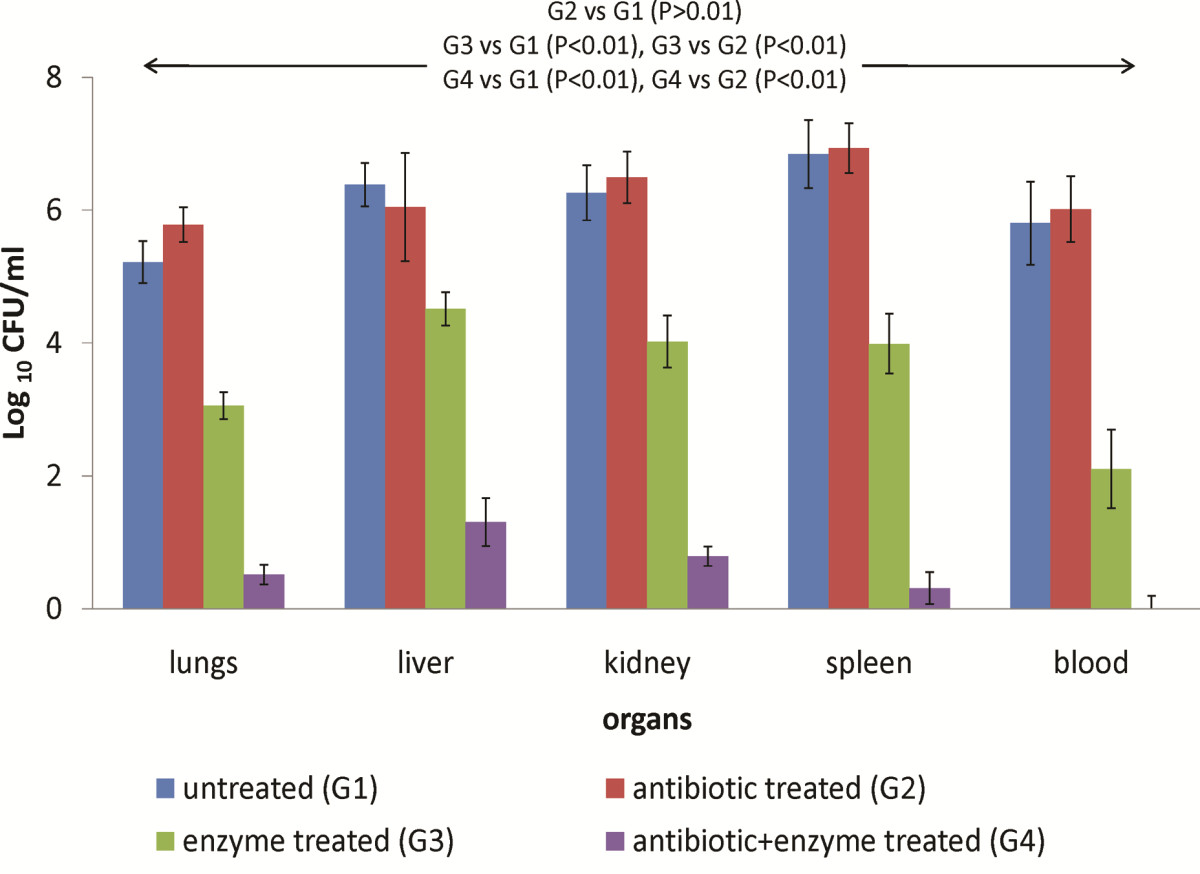
**Bacterial load (Log**_**10**_ **CFU/ml) 24 h post infection in various organs and blood of mice infected via intraperitoneal route with** ***K. pneumoniae*** **B5055 and treated with gentamicin (1.5 mg/kg) and/or depolymerase (50 μg).** G1, G2, G3, G4 represent Groups 1, 2, 3, 4. Arrows indicate significant reduction in Log bacterial count in all organs and blood from mice belonging to different groups. Error bars represent standard deviation (S.D) from four independent values.

Intraperitoneal administration of 10^2^ CFU/0.1 ml of *K. pneumoniae* in mice led to bacterial colonization of liver(s) (6.045 ± 0.325), lungs (5.778 ± 0.314), kidney(s) (6.491 ± 0.415), spleen(s) (6.934 ± 0.512) and bacteremia (6.011 ± 0.624) within 24 h (Figure 2). Owing to a massive spread of bacteria via systemic circulation, administration of gentamicin 6 h later did not provide any significant protection (P > 0.01). In contrast, intraperitoneal administration of a single 50 μg dose of bacterial depolymerase, 6 h post infection resulted in significant reductions (P < 0.01) of ~2.7 logs, ~1.5 logs, ~2.5 logs, ~ 2.94 logs and ~3.9 logs in bacterial counts in lungs, liver(s), kidney(s), spleen(s) and blood respectively (Figure 2). Simultaneous administration of gentamicin and depolymerase, led to almost complete eradication of bacteria, abolished bacteremia and prevented bacterial dissemination into various organs.

### Effect of intra-peritoneal administration of bacterial depolymerase on cytokine expression during compartmentalized and systemic infection

**Figure 3:**
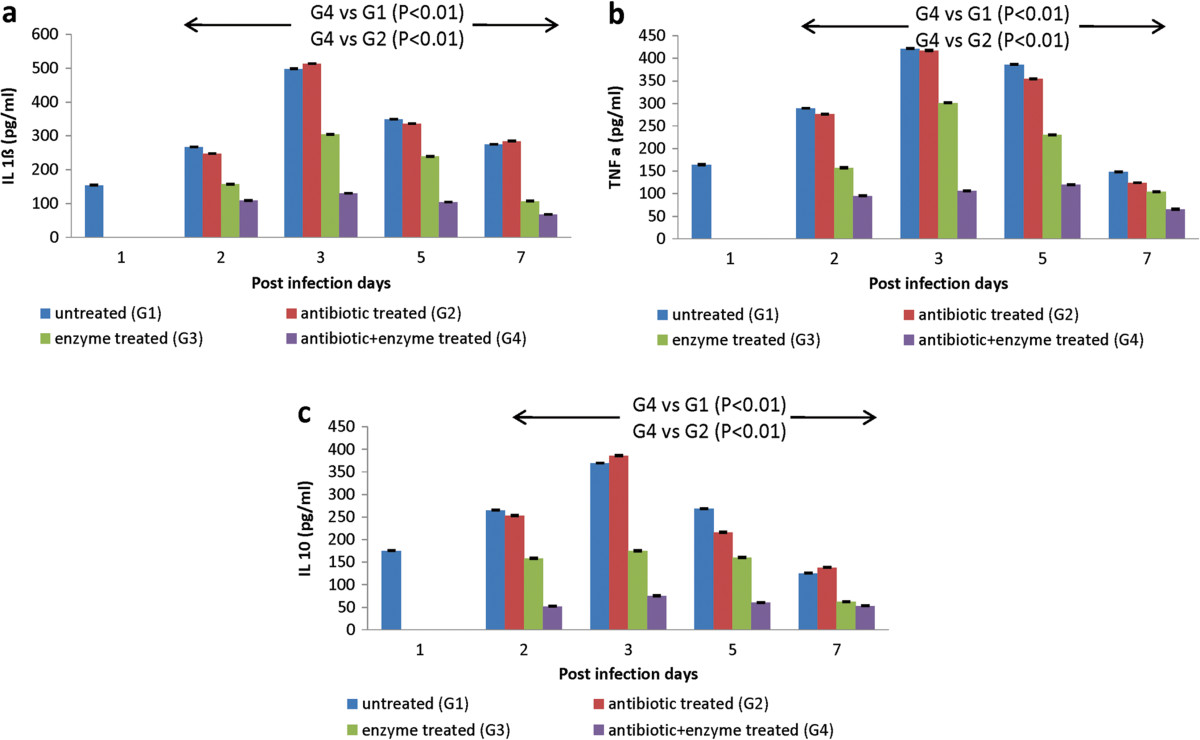
**Cytokine levels during acute lung infection: a) IL 1ß b) IL 10 c) TNF** ***a*** **levels (pg/ml) in lungs of mice infected via intranasal route with** ***K. pneumoniae*** **B5055 and treated with gentamicin (1.5 mg/kg) and/or depolymerase (50 μg).** No sample was taken and processed on day 1 in the treated groups. G1, G2, G3, G4 represent Groups 1, 2, 3, 4. Arrows indicate significant reduction in cytokine levels between groups on days 2, 3, 5, 7. Error bars represent standard deviation (S.D) from four independent values.

Acute lung infection with *K. pneumoniae* accentuated the release of IL- 1ß, IL-10 and TNF*a* in untreated animals (Figure 3). Administration of bacterial depolymerase alone or in combination with gentamicin resulted in significant reduction (P < 0.01) in cytokine expression. In comparison to the uninfected or antibiotic treated animals, a decrease of ~1.63 fold (IL-1ß), ~1.39 fold (TNF*a*) and ~2.0 fold (IL-10) was observed in depolymerase alone treated mice on day 3. In contrast, a significant reduction (P < 0.01) of ~3.8 folds (IL-1ß), ~3.97 folds (TNF*a*) and ~4.9 folds (IL-10) was seen in depolymerase + gentamicin treated group (Figure 3a, b, c) on peak day of infection (day 3).

**Figure 4:**
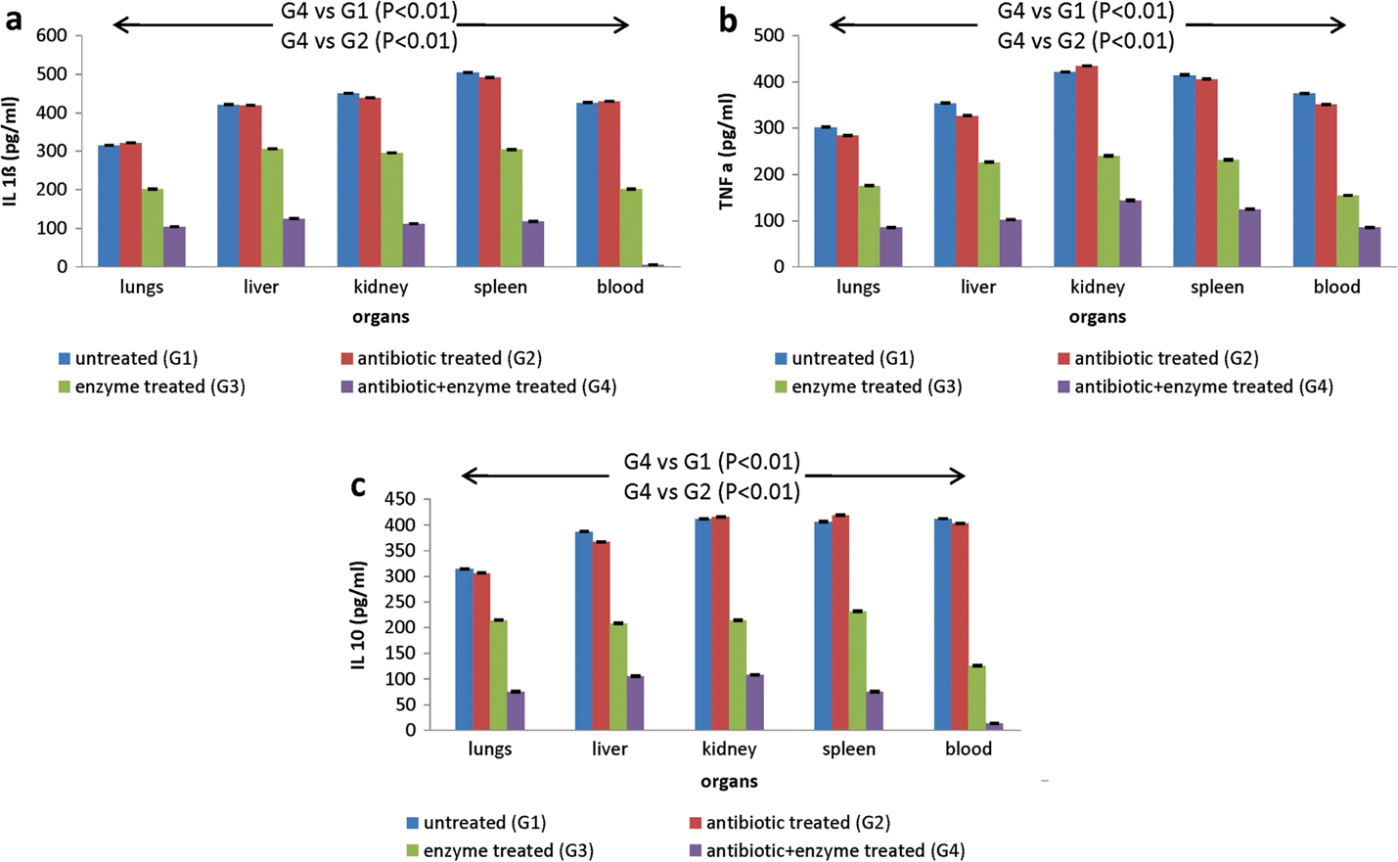
**Cytokine levels during septicemia: a) IL 1ß b) IL 10 c) TNF** ***a*** **levels (pg/ml) 24 h post infection in various organs and blood of mice infected via intra peritoneal route with** ***K. pneumoniae*** **B5055 and treated with gentamicin (1.5 mg/kg) and/or depolymerase (50 μg).** G1, G2, G3, G4 represent Groups 1, 2, 3, 4. Arrows indicate significant reduction in cytokine levels in all organs and blood from mice belonging to different groups. Error bars represent standard deviation (S.D) from four independent values.

In animals receiving infection intraperitoneally, systemic spread of infection resulted in marked upregulation in pro-inflammatory as well as anti-inflammatory cytokine levels within 24 h. A modest downregulation in levels of IL-1ß [~1.56 fold (lungs), ~1.37 fold (liver), ~1.52 fold (kidney), ~1.65 fold (spleen)], TNF*a* [~1.72 fold (lungs), ~1.56 fold (liver), ~1.75 fold (kidney), ~1.79 fold (spleen)] and IL-10 [~1.46 fold (lungs), ~1.86 fold (liver), ~1.92 fold (kidney), ~1.75 fold (spleen)] was observed in the infected, enzyme alone treated animals (Figure 4a, b, c). In contrast, cytokine levels in the blood serum samples were significantly reduced (P < 0.01) by ~2.1 fold (IL-1ß), ~2.43 fold (TNF*a* and ~3.26 fold (IL-10) in this group. When depolymerase was used along with gentamicin, negligible levels of all cytokines (P < 0.01) were observed in lungs, liver, kidney, spleen and blood (Figure 4a, b, c).

### Histopathological analysis

**Figure 5:**
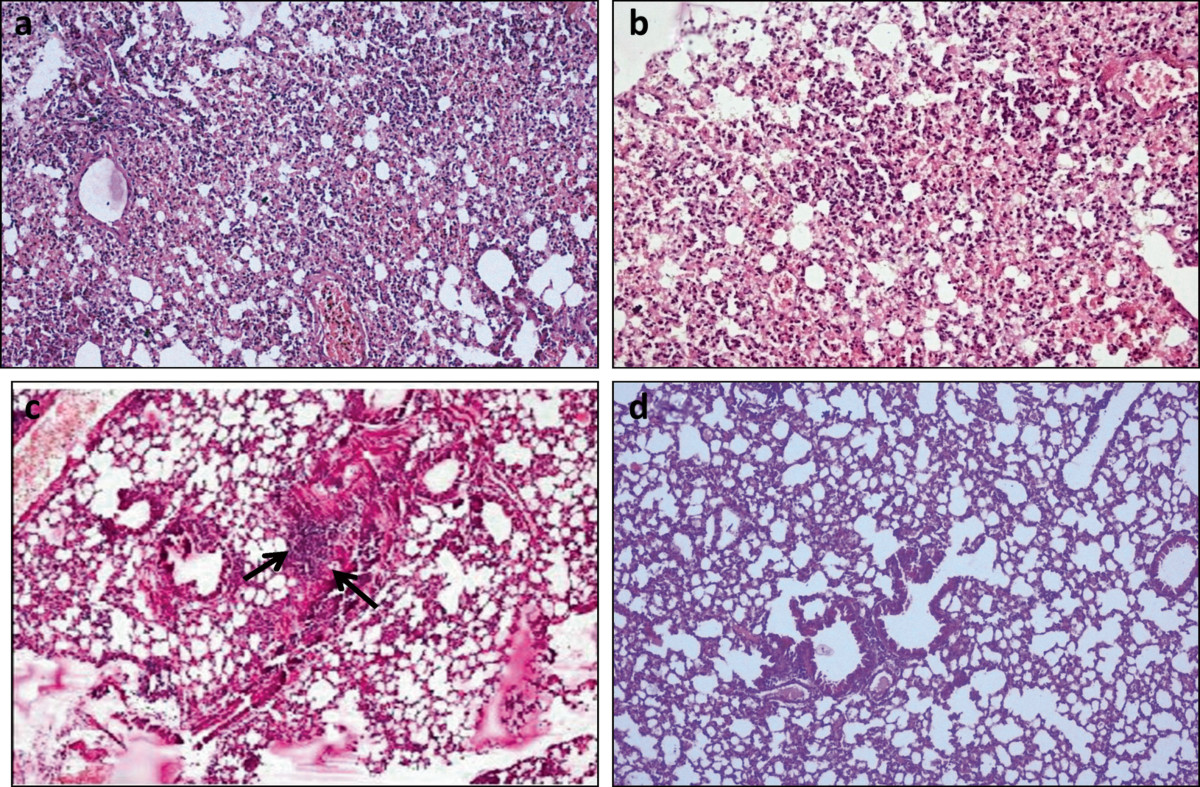
**Lungs of mice infected with** ***K. pneumoniae*** **B5055 and given various treatments intraperitoneally, 24 h post infection (a) normal saline alone, i.e. control group. (b)** gentamicin treated **(c)** bacterial depolymerase treated (arrows indicate peribronchial inflammation) **(d)** bacterial depolymerase and gentamicin treated. (Magnification: 100×).

Massive infiltration of alveoli with neutrophils was observed in lungs of control (Figure 5a) and gentamicin treated mice (Figure 5b) harvested on the peak day (day 3) of infection. This indicated the inability of antibiotic to control the exaggerated state of inflammation in response to infection. In contrast, depolymerase treated animals although showed signs of peribronchial inflammation but had no neutrophil infiltration on day 3 (Figure 5c). Combination treated mice exhibited no signs of inflammation or neutrophil extravasation (Figure 5d).

### Protective efficacy of enzyme in animals previously exposed to depolymerase

**Figure 6:**
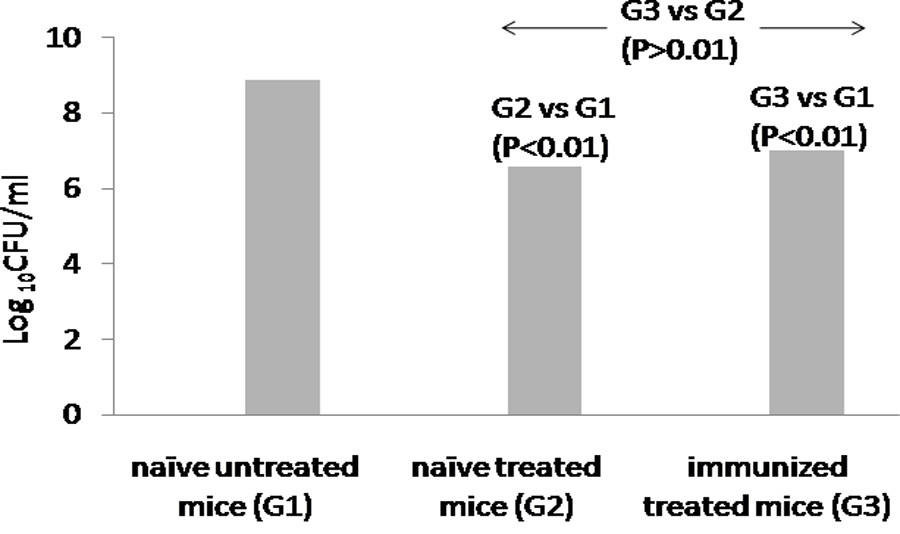
**Log**_**10**_ **CFU/ml on 3** ^**rd**^ **day in naïve untreated mice (Group 1, G1), naïve treated mice (Group 2, G2) and immunized treated mice (Group 3, G3) challenged with 10** ^**4**^ **CFU/50 μl** ***K. pneumoniae*** **B5055.**

To address the possibility of antibodies against depolymerase, neutralizing the protective efficacy offered by it, hyperimmune serum was raised against the purified protein in mice. Antibody titer of 1,000 was obtained against depolymerase. Thereafter, mice with antisera against depolymerase were challenged intranasally with 10^4^ CFU/50 μl. These were then treated with 50 μg of depolymerase, 24 h post infection. A count of 8.9 Log_10_CFU/ml was observed in naïve infected untreated mice on the peak day (day 3) (Figure 6). In contrast, no significant difference (P > 0.01) in bacterial count was observed in immunized treated mice (7 Log_10_CFU/ml) and naïve treated mice [(6.6 Log_10_CFU/ml), not previously exposed to the enzyme hence with no antisera] (Figure 6).

**Figure 7:**
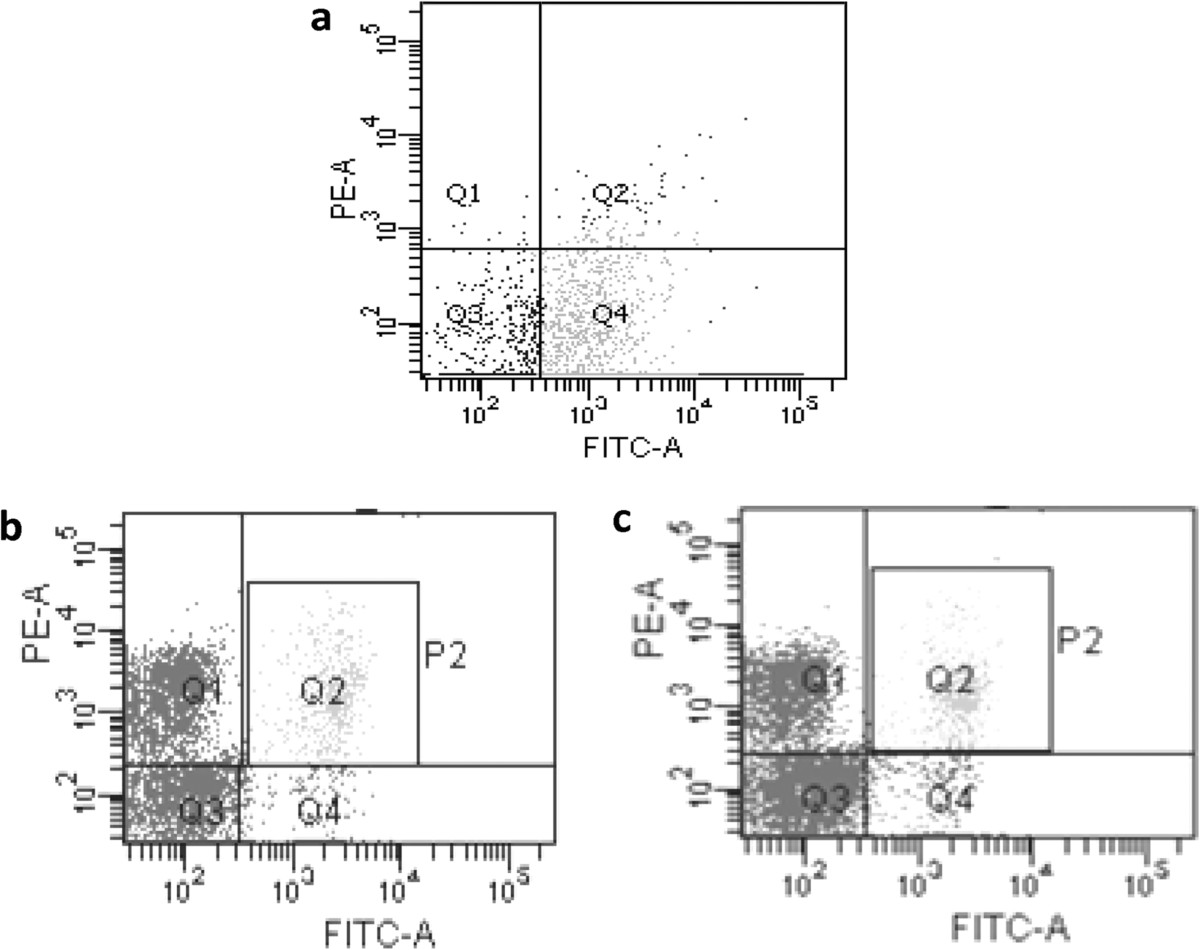
**Phagocytic killing of untreated bacteria (a) bacteria treated with depolymerase pre-incubated with naïve sera (b) bacteria treated with depolymerase pre-incubated with immune sera (c) as determined through flow cytometry after staining with live-dead staining kit.** (Q4:live cells, Q1:dead cells).

Furthermore, to confirm the retention of catalytic activity by bacterial depolymerase after pre-incubation with antisera raised against it, percentage killing of *Klebsiella* by the mouse peritoneal macrophages was estimated. As observed by flow-cytometry, percentage macrophage killing of bacteria treated with depolymerase pre-incubated with antisera was 93.6% (Figure 7c, Q1). Similarly, bacteria which were treated with enzyme pre-incubated with naïve sera were 95% dead (Figure 7b, Q1). This indicated no significant difference in percentage killing of either group of bacteria by the macrophages after 3 h. In contrast, a significantly less percentage macrophage killing was observed for the untreated bacteria (10%, Figure 7a, Q1).

## Discussion

*K. pneumoniae* accounts for 25-43% of nosocomial pneumonias caused by gram negative bacteria. It has a rapid progressive clinical course often complicated by multilobular involvement and lung abscesses [23] which leaves little time to institute effective antimicrobial treatment. As a result, the mortality rates may reach or exceed 50% even in treated cases [24]. The voluminous capsular layer is involved in adhesion, maintenance, proliferation and development of infections by this pathogen [25]. Owing to an increase in the MDR and NDM-1 strains, the WHO recommended ampicillin and gentamicin as first line of treatment have proved to be ineffective [26]. Thus a demand for newer antimicrobials, not affected by resistance mechanisms has gained momentum in the last decade [27].

Promising results obtained after using *A. punctata* derived depolymerase that lyses K2 capsule of *K. pneumoniae,* thereby improving gentamicin efficacy against planktonic and biofilm cells of *K. pneumoniae* (data not presented) during *in vitro* studies, prompted us to evaluate its *in vivo* therapeutic efficacy. Post infection administration of single bolus of bacterial depolymerase, resulted in significant drop (~2 log) in bacterial titers during pulmonary infection. Kabha *et al.,*[28] have reported that *Klebsiella* with certain CPS types like K21a, containing mannose-*a*-2-mannose and rhamnose-*a*-2/3-rhamnose sequences are readily recognized by the macrophage mannose receptor followed by their ingestion and killing. In contrast, strains with K2 CPS lacking these sequences are not recognized, thus allowing expression of virulence factors and bacterial proliferation in various organs. Denudation of K2 capsule by *Aeromonas* derived enzyme possibly led to improved uptake and killing of *Klebsiella* by alveolar macrophages in lungs.

Studies by Hoffman et al. [29] and Durante-Mangoni et al. [30] have reported wide use of aminoglycosides to treat bacterial infections of heart, lung and urinary tract. Thus, gentamicin was chosen for treating *K. pneumoniae* induced acute lung infection. When used at 2.5 mg/kg or 5 mg/kg post infection, highly significant reduction (P < 0.001) in bacterial load was observed (data not presented) indicating its high efficacy at these concentrations. When 1.5 mg/kg gentamicin was administered at 0 h, 6 h, 12 h, 24 h, 48 h, 24 h + 48 h post infection, significant reduction in bacterial load was observed in the first 3 groups on peak day (day 3) (data not presented). But in the other groups, gentamicin (1.5 mg/kg) alone could not control the infection. Even when injected daily till 7^th^ day post infection, no significant protection was observed. These results indicated that gentamicin was effective during the initial time when bacteria have not completely established themselves or started to proliferate in the lung tissue. Once bacteria colonized, proliferated in the lung and CPS production was maximal, gentamicin was no longer effective. Moreover, in clinical situations also, it takes some time to initiate antibiotic treatment. Furthermore, Lavender et al. [31], have reported that early stages of *K. pneumoniae* airway infections might involve biofilm formation. As suggested by Kristian et al. [32], biofilm formation inhibits effectiveness of antibiotic treatment, prevents deposition of host defense components including C3b and IgG and facilitates bacterial communication leading to expression of virulence determinants. Therefore, *A. punctata* derived depolymerase was co-injected 24 h post infection with gentamicin to check whether it could remove CPS and render the bacteria susceptible to gentamicin. A reduction of ≥99% in bacterial titers was seen. This can be attributed to the enzyme mediated dispersal of CPS matrix leading to improved susceptibility of bacteria towards an otherwise ineffective gentamicin concentration. Depolymerase and gentamicin were comparatively less effective on the 3^rd^ day because bacterial infection and tissue injury were at its peak and they were unable to tackle it on their own. Since the infection was confined to lung only, hence, as the bacterial number decreased by day 4/5, the agents became more effective. Thus enzyme based therapy helped to overcome the limitations offered by CPS including slow penetration of aminoglycosides due to electrostatic interactions with mucus and biofilm matrix. Disruption of capsular layer also possibly led to improved opsonization and increase in effector function of leukocytes leading to significant reduction in bacterial count. Reports by Brown, [33], using antibiotics and mucolytics in cystic fibrosis patients have also suggested that the latter provided symptomatic relief by decreasing mucus viscosity, thereby facilitating bacterial clearance.

Spread of bacteria on intraperitoneal administration led to bacteremia and colonization of liver, kidney, spleen and lungs. After intraperitoneal administration, the enzyme gained quick access to these organs via systemic circulation and through capsule removal facilitated bacterial clearance by mononuclear phagocytic system operating in these organs. Merino et al. [34] suggested that during planktonic growth, K2 capsule of *Klebsiella* causes complement activation, but activated complement components bind far from cell membrane hence, no cell lysis occurs. Highly significant reduction of bacterial count in blood on treatment of mice with enzyme can be attributed to capsule removal which facilitated deposition of complement components resulting in cell death. Decapsulating enzymes like endosialidase against *E. coli* K1 and poly-glutamic acid depolymerase against *B. anthracis* have been shown to prevent the spread of infections by these bacteria in experimental animals by enhancing their killing by complement, neutrophils and macrophages [35–39]. Moreover, reports showing synergy between enzyme and antibiotic as observed in this study, have also appeared previously in Gram positive infections caused by *Staphylococcus* and *Pneumococcus*[40, 41].

During acute lung infection, bacterial components (CPS and LPS), macrophage and neutrophil mediators (oxygen radicals, proteolytic enzymes) and complement components induce an inflammatory response. Thus, in the present study significant increase was observed in levels of pro-inflammatory as well as anti-inflammatory cytokines during compartmentalized pneumonia. Witzenrath et al. [42] and Zelmer et al. [43] have reported reduction in cytokine expression after treatment of *Streptococcus pneumoniae* infection with lytic enzyme (Cpl-1) and *E. coli* K1 infection with endosialidase (endoE). Similarly, in our study significant reduction in cytokine expression was observed in treated animals. It protected mice from pathogen induced damage and helped in clearance of invading bacteria. In enzyme treated animals, during systemic infection, reduction in cytokine expression was highly significant, in comparison to that observed during compartmentalized pneumonia. This indicates that during respiratory infection, enzyme although interrupts the course of infection, but does not completely diminish, local tissue response to bacterial invasion. This might be due to extensive proliferation of the pathogen on mucosal layer of respiratory tract in biofilm mode contrary to the presence of planktonic bacteria during systemic spread. Histopathological examination of lung tissue of infected, untreated animals showed well-developed pneumonia with neutrophil infiltration, abscess formation and destruction of alveoli as previously described in our laboratory [21]. In contrast, lung tissue of mice treated with enzyme alone showed signs of peribronchial inflammation but lung alveoli were completely devoid of neutrophils. On the other hand, no signs of inflammation and neutrophil extravasation was observed in mice treated with enzyme and gentamicin. Thus it could be concluded that, enzyme mediated capsule removal did not allow high bacterial density to be reached, reduced the residence time of bacteria in mice and sensitized decapsulated bacteria to gentamicin and components of the immune system.

Since, proteins are immunogenic when delivered systemically, therefore the issue of neutralizing antibodies interfering with activity of depolymerase after its *in vivo* administration was addressed. Inspite of the presence of antibodies, the ‘enzybiotic’ was equally active in immunized and naïve mice. Moreover, pre-incubation of enzyme with its antisera did not hinder the overall bacterial killing by immune cells. This might be due to presence of antibodies directed against epitopes that do not contribute to therapeutic potential of protein or due to a higher binding affinity of the enzyme for its substrate in comparison to the antibody’s affinity for enzyme [44].

## Conclusion

Conventional antibiotic therapy during gram negative infections generally involves treatment with broad spectrum antibiotics especially combination of aminoglycoside and third-generation cephalosporins [45]. But, poor antibiotic efficacy, emergence of resistant variants and side effects limit their use *in vivo*. In contrast, enzymes do not allow development of resistance, improve antimicrobial efficacy and have no untoward effects. Reassuring results obtained after *in vivo* administration of bacterial depolymerase, warrant its further examination before including this strategy in the routine treatment regime. With, protein engineering, domain swapping and gene shuffling one can even evolve a better enzyme for controlling bacterial pathogens in clinical settings.

## Electronic supplementary material

Additional file 1: **Microscopic appearance of** ***K. pneumoniae*** **B5055: encapsulated untreated bacteria (a) depolymerase treated bacteria (b).** The bacterial cells were taken on a clean glass slide. A drop of safranin was mixed with bacterial culture and the suspension was spread neatly on the slide. The smear was stained with crystal violet for 1 min, washed gently and observed under a light microscope (40X). (PDF 144 KB)

Below are the links to the authors’ original submitted files for images.

Authors’ original file for figure 1

Authors’ original file for figure 2

Authors’ original file for figure 3

Authors’ original file for figure 4

Authors’ original file for figure 5

Authors’ original file for figure 6

Authors’ original file for figure 7
