# Supplementary material for: Depolymerase improves gentamicin efficacy during Klebsiella pneumoniae induced murine infection
Source: BMC Infect Dis. 2014 Aug 23;14:456. doi: 10.1186/1471-2334-14-456 (PMC4150946; doi:10.1186/1471-2334-14-456)
Supplement: Supplementary file 1 — Additional file 1: Microscopic appearance of K. pneumoniae B5055: encapsulated untreated bacteria (a) depolymerase treated bacteria (b). The bacterial cells were taken on a clean glass slide. A drop of safranin was mixed with bacterial culture and the suspension was spread neatly on the slide. The smear was stained with crystal violet for 1 min, washed gently and observed under a light microscope (40X). (PDF 144 KB) [file 12879_2014_3750_MOESM1_ESM.pdf]

Additional file 1

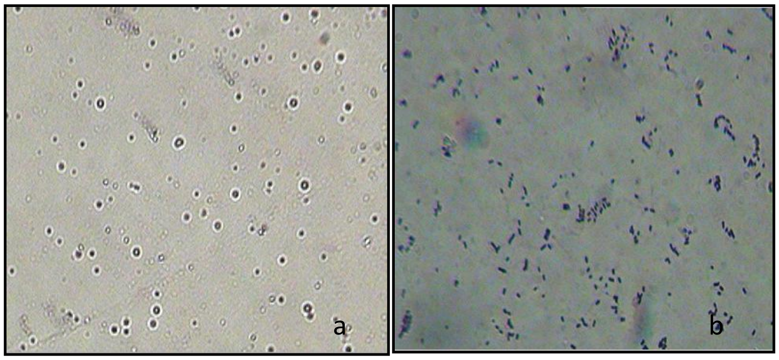

Microscopic appearance of *K. pneumoniae* B5055: encapsulated untreated bacteria (a) depolymerase treated bacteria (b). The bacterial cells were taken on a clean glass slide. A drop of safranin was mixed with bacterial culture and the suspension was spread neatly on the slide. The smear was stained with crystal violet for 1 min, washed gently and observed under a light microscope (40X).
