# Supplementary figures and images for: Depolymerase improves gentamicin efficacy during Klebsiella pneumoniae induced murine infection
Source: BMC Infect Dis. 2014 Aug 23;14:456. doi: 10.1186/1471-2334-14-456 (PMC4150946; doi:10.1186/1471-2334-14-456)

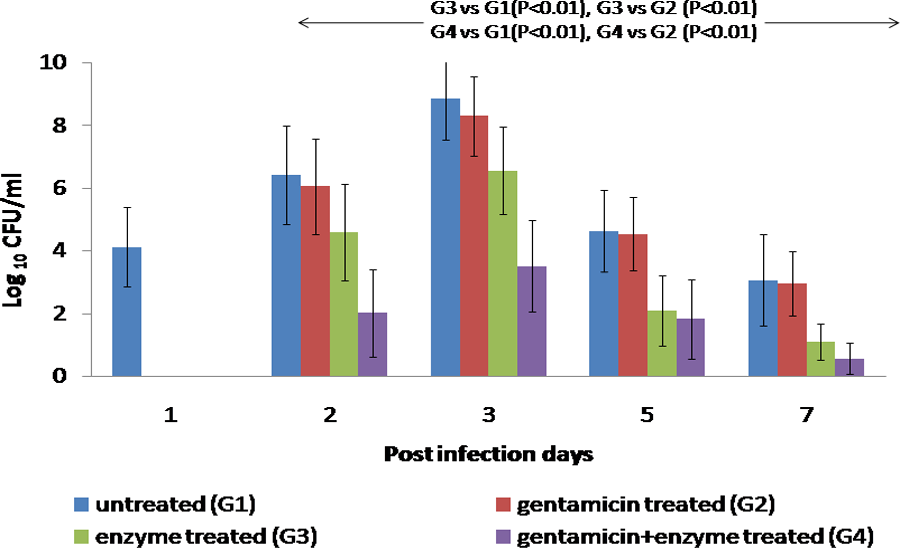

Supplement: Supplementary file 2 — Authors’ original file for figure 1 [file 12879_2014_3750_MOESM2_ESM.tiff]

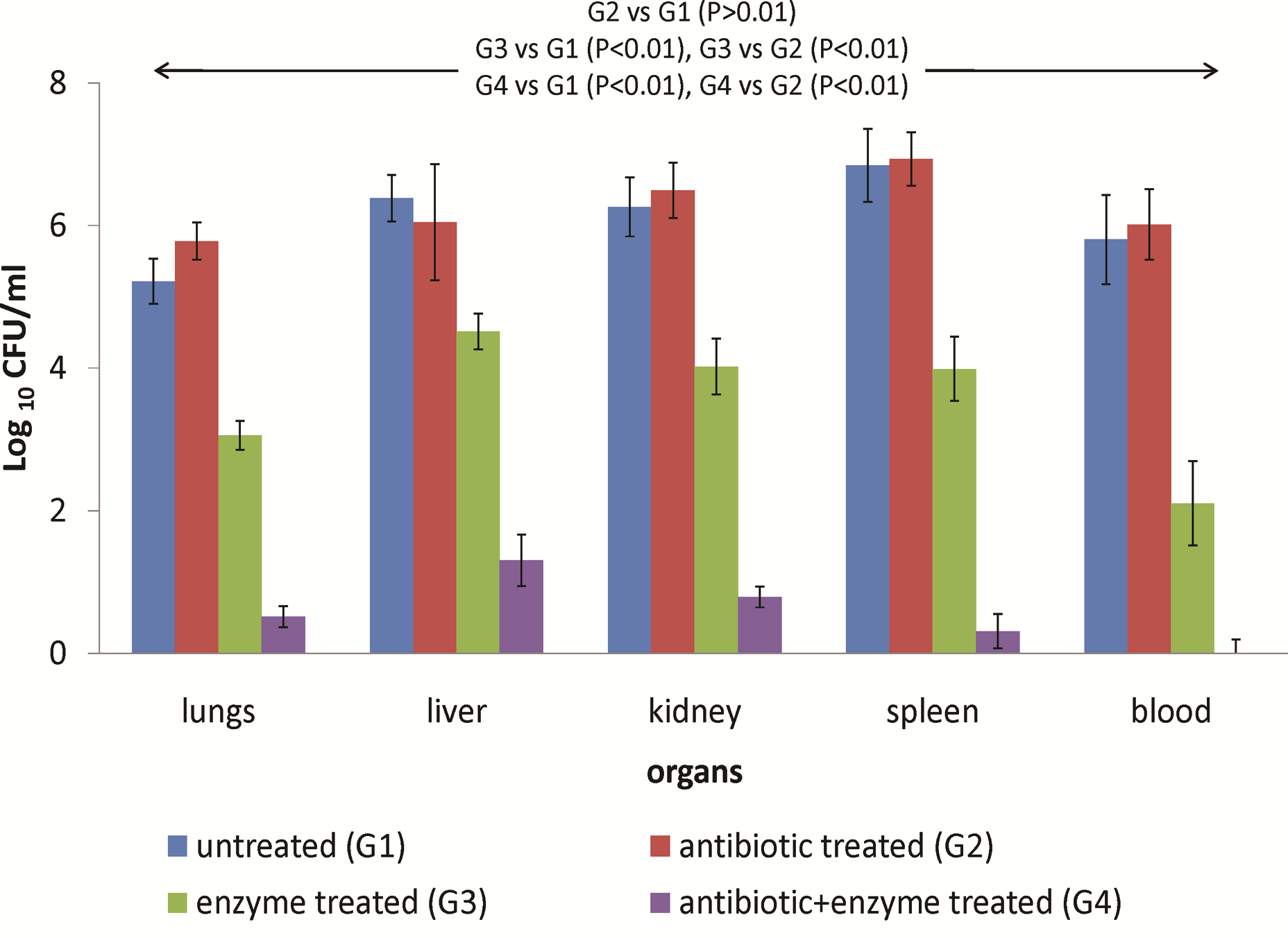

Supplement: Supplementary file 3 — Authors’ original file for figure 2 [file 12879_2014_3750_MOESM3_ESM.tiff]

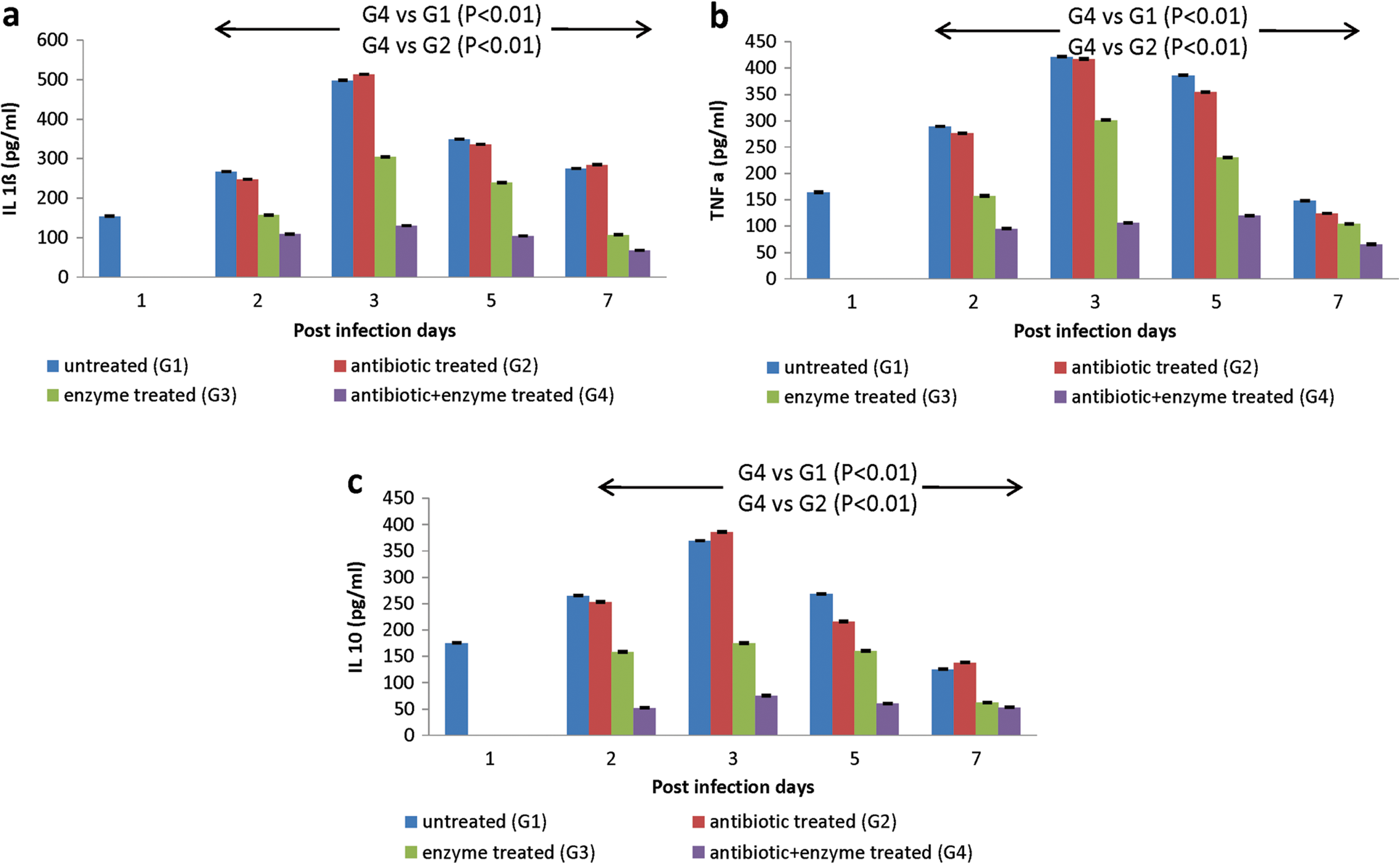

Supplement: Supplementary file 4 — Authors’ original file for figure 3 [file 12879_2014_3750_MOESM4_ESM.tiff]

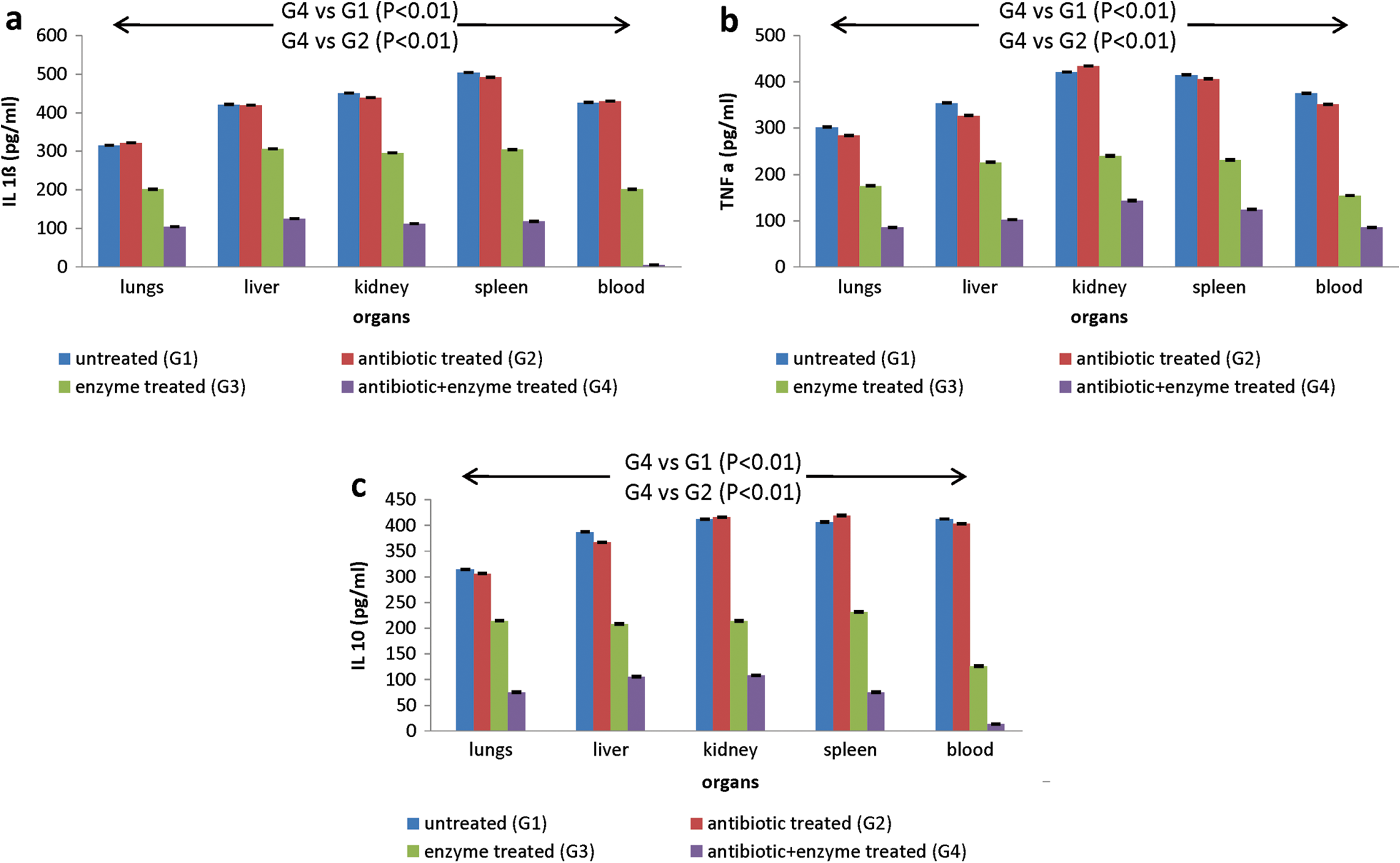

Supplement: Supplementary file 5 — Authors’ original file for figure 4 [file 12879_2014_3750_MOESM5_ESM.tiff]

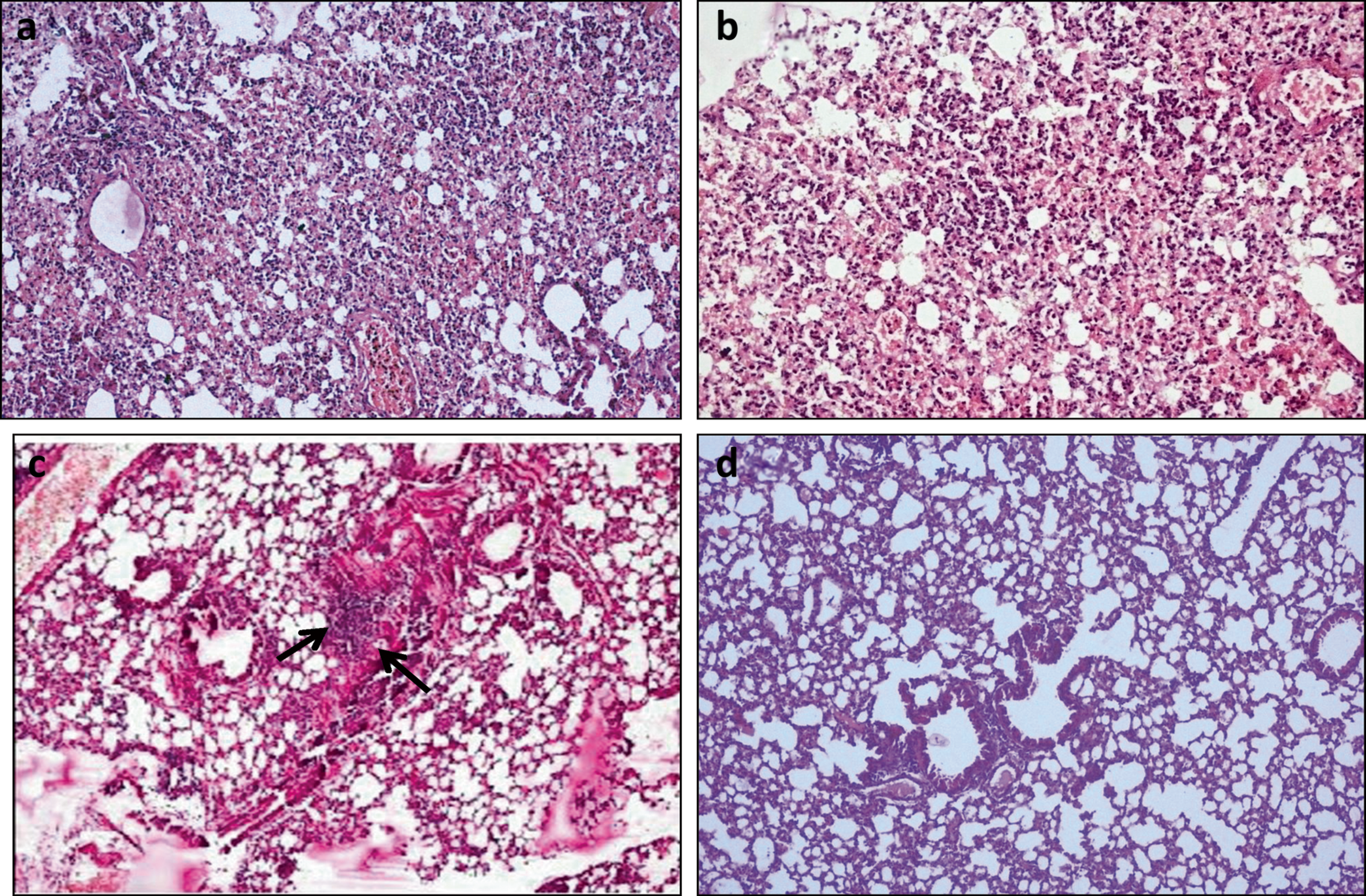

Supplement: Supplementary file 6 — Authors’ original file for figure 5 [file 12879_2014_3750_MOESM6_ESM.tiff]

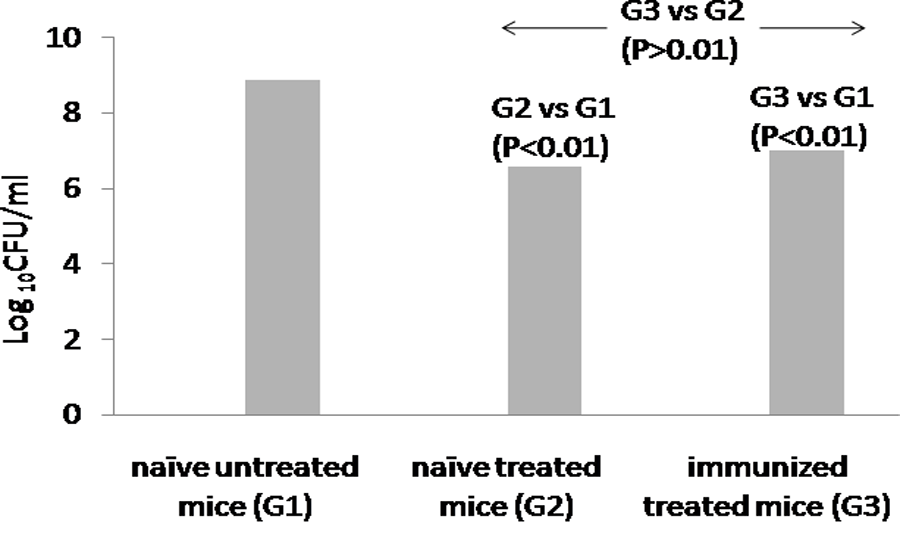

Supplement: Supplementary file 7 — Authors’ original file for figure 6 [file 12879_2014_3750_MOESM7_ESM.tiff]

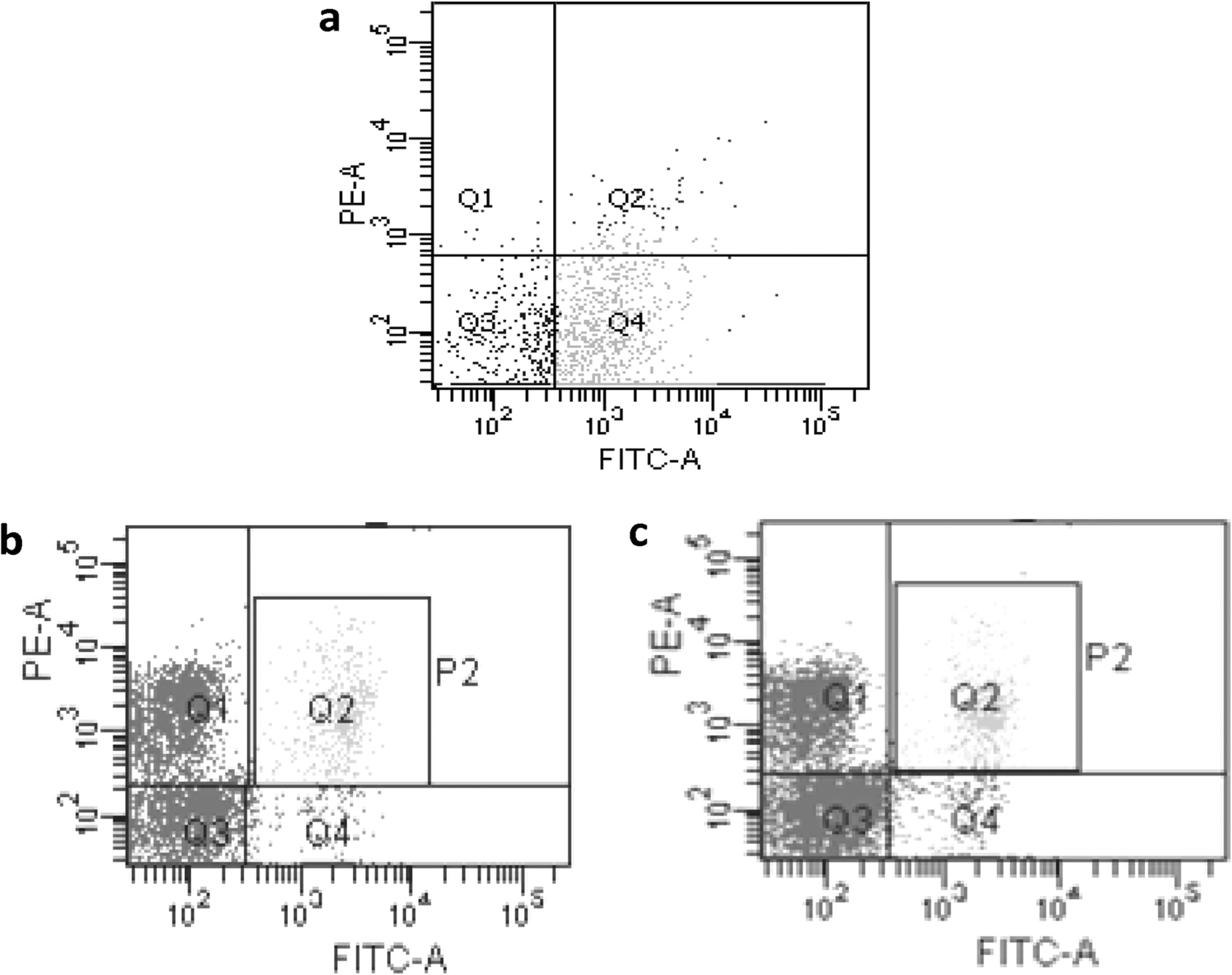

Supplement: Supplementary file 8 — Authors’ original file for figure 7 [file 12879_2014_3750_MOESM8_ESM.tiff]
